# Research Status of Lignin-Based Polyurethane and Its Application in Flexible Electronics

**DOI:** 10.3390/polym16162340

**Published:** 2024-08-19

**Authors:** Jingbo Hu, Mengmeng Huang, Xing Zhou, Rubai Luo, Lu Li, Xiaoning Li

**Affiliations:** 1 Faculty of Printing, Packaging Engineering and Digital Media Technology, Xi’an University of Technology, Xi’an 710048, China; huangmm921@163.com (M.H.); luorubai@xaut.edu.cn (R.L.); 15518872069@163.com (X.L.); 2 Key Laboratory of Auxiliary Chemistry and Technology for Chemical Industry, Ministry of Education, Shaanxi University of Science and Technology, Xi’an 710021, China; lilu@sust.edu.cn; 3 Shaanxi Collaborative Innovation Center of Industrial Auxiliary Chemistry and Technology, Shaanxi University of Science and Technology, Xi’an 710021, China

**Keywords:** polyurethanes, lignin, green chemistry, polyol, flexible electronics

## Abstract

Polyurethanes (PU) have drawn great attention due to their excellent mechanical properties and self-healing and recyclable abilities. Lignin is a natural and renewable raw material in nature, composed of a large number of hydroxyl groups, and has a great potential to replace petroleum polyols in PU synthesis. This review summarizes the recent advances in modification methods such as the liquefaction, alkylation, and demethylation of lignin, and a systematic analysis of how to improve the reactivity and monomer substitution of lignin during polyurethane synthesis for the green manufacturing of high-performance polyurethanes was conducted. Polyurethane can be used in the form of films, foams, and elastomers instead of conventional materials as a dielectric or substrate material to improve the reliability and durability of flexible sensors; this review summarizes the green synthesis of polyurethanes and their applications in flexible electronics, which are expected to provide inspiration for the wearable electronics sector.

## 1. Introduction

Conventional polyurethanes (PU) are obtained via the addition reaction of polyols and isocyanates, and have been widely used in foams [1,2,3], elastomers [4,5], coatings [6], adhesives [7,8,9,10], and other fields. However, most of the polyols required for the preparation of polyurethanes come from non-renewable petroleum resources. It is important to prepare PU from raw environmental materials, using methods such as recycling and renewable polymers, bio-based materials, and even biomacromolecules [11,12,13]. In addition, polyurethane is widely used in flexible substrates for electronic devices due to its excellent properties. According to Statista, the global electronic devices market generated a total revenue of approximately USD 2.98 trillion in 2021 and is expected to reach USD 3.2 trillion by 2024. Therefore, among the potential raw materials for PU, lignin is currently a hot topic for researchers due to its easy availability [14], low cost [15], and molecular composition, leading to the potential application of lignin-based polyurethanes for flexible electronics.

Lignin, one of the main components of plant cell walls, is an amorphous polymer consisting of benzene and propane units linked by carbon–carbon and ether bonds [16,17,18]. It is a potential substitute for petroleum resources due to its rich natural aromatic content. This reduces dependence on non-renewable resources and lowers the impact on the environment. Lignin can be used in the synthesis of polyurethanes through direct incorporation [19]. In addition to lignin, isocyanate groups can also be reacted with cellulose [20] and cellulose nanocrystals [21] to, for example, synthesize bio-based polyurethanes. However, the complex and branched molecular structure of lignin makes it less reactive towards isocyanates [22]. Therefore, lignin can be modified so that reactive groups in the lignin structure [23,24], such as phenolic hydroxyls and alcohol hydroxyls, can partially or completely replace hydroxyl groups in reactive polyols and participate in the synthesis of lignin-containing polyurethane materials. In addition, lignin can be added to polyurethane materials as an active filler by reacting with isocyanates [25,26,27].

Extensive research efforts have been devoted to increasing the application potential of lignin by employing a variety of countermeasures including hydrolysis, pyrolysis, and depolymerization [28]. In recent years, the application of lignin to polyurethanes through chemical modification has become a prominent area of research in the field of lignin value addition and has attracted much attention. Several methods have been developed, ranging from fractionation to sulfonation modification, amination, and anti-solvent methods. For example, Guo [29] and Li et al. [30] fractionated lignin with organic solvents, effectively reducing the heterogeneity of lignin and preparing polyurethane foams with excellent properties using different fractions of lignin. At the same time, it has been shown that polyurethane foams containing lignin degrade at a rate about 2.6 times faster than lignin-free foams [31]. This accelerated degradation performance helps to reduce the accumulation of plastic waste in the environment, thus making it more environmentally friendly. Fan et al. [32] used an anti-solvent method to address the disadvantages of the non-durability of WPUs, solving the drawback of WPUs’ lack of durability. Li et al. [33] pretreated lignin with recoverable ethylenediamine (EDA) and showed better thermal stability. In addition, by changing the synthesis idea of traditional polyurethanes and adopting the synthesis based on the isocyanate index of the lignin hydroxyl group being less than 1.0, the excess hydroxyl group in lignin is exchanged with the carbamate bond to form a dynamic covalent network [34]. The advantage of this method is that it reduces the amount of isocyanate, which is conducive to improving the environmental friendliness of polyurethane materials. Despite these advances, it is still challenging to prepare homogeneous and highly reactive lignin in a relatively simple manner and to apply it effectively in polyurethanes.

Based on the above aspects, the aim of this paper is to outline the value of lignin in the preparation of polyurethanes. Specific methods for modifying lignin and synthesizing green bio-based PUs in recent years are summarized to explore the development of their applications in recyclable, self-repairing, and reconfigurable flexible polyurethane materials, as well as the future prospects of wearable electronic devices.

## 2. Synthesis of Polyurethane

**Figure 1:**
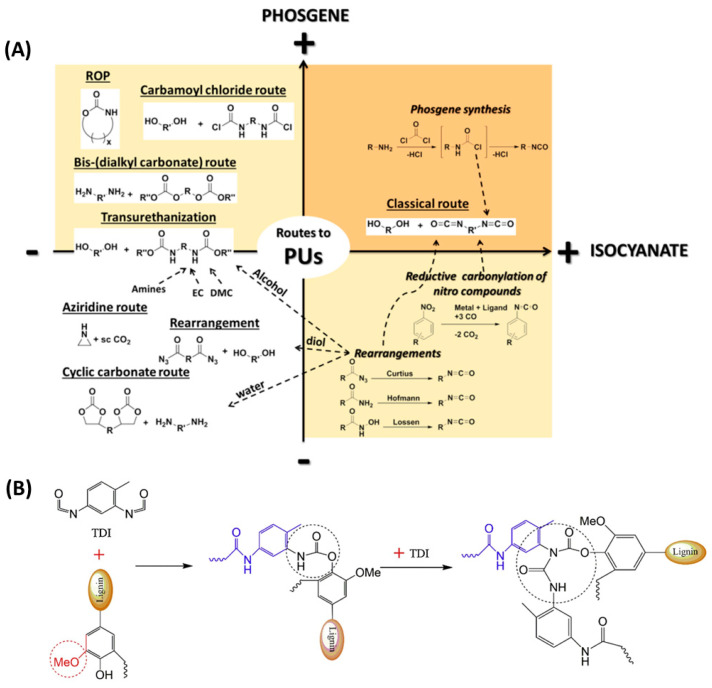
PU synthesis path. (**A**) Overview of the main synthetic routes to Pus [35]. (**B**) Reaction mechanism of lignin and diisocyanate (diisocyanate takes TDI (Toluene-2,4-diisocyanate) as an example) [39].

Numerous synthesis methods of PUs have been investigated (Figure 1A) [35]. One of which aims to achieve an addition reaction between a polyol and isocyanate, which mainly focuses on the high reactivity of the isocyanate group -NCO towards unstable hydrogens on the alcohol compound. Another method is a one-step process: mix oligomers, diisocyanatos, chain extenders, and catalysts simultaneously, inject the mixture into the mold, and then cure at a certain temperature [36]. In the synthesis of PUs, hydroxy polyester or polyester polymer polyols form the soft segments, while aromatic or aliphatic diisocyanatos or isocyanates form the hard segments of PUs. Based on the interaction of hydrogen bonds between urethanes, soft and hard segments can form microphase zones and produce microphase separation, giving polyurethane superior wear resistance and toughness. By using different types of diols and isocyanates as raw materials, thermoplastic and thermosetting PUs with different soft and hard segment structures can be obtained. Therefore, the properties of PUs can be modified by adjusting the property, structure, and molar mass of the polyol and the isocyanate used during the synthesis process [37].

On the environmental front, polyurethane raises concerns in the manufacturing process. Most of the polyols used in the synthesis of PUs come from non-renewable petroleum resources and are poorly biodegradable, leading to soil and ecological decontamination. With regard to safety, most of the chemicals used in the synthesis of polyurethanes, such as isocyanates and dibutyltin dilaurate (DBTDL), are considered to be carcinogenic at low concentrations or as inhalation toxicants. To achieve the goal of sustainable development, it is necessary to come up with a new approach using renewable resources [38]. Lignin, as a natural and renewable aromatic polymer compound, has the advantages of abundant sources, renewability, and low price. Its molecular structure contains both aromatic and hydroxyl functional groups. As lignin contains a large number of hydroxyl groups, it can react with isocyanate groups to form polyurethane prepolymers, which can be polymerized to form polyurethane. It usually needs to be chemically modified into polyols for the synthesis of polyurethanes. As listed in Table 1, the main preparation methods, materials, and the PU properties are presented and compared. Figure 1B presents the reaction mechanism of lignin and diisocyanate [39]. Polyurethane is synthesized through a two-step reaction, where hydroxyl groups in lignin react with isocyanates to form amino ester bonds.

## 3. Methods for Preparing Polyols from Lignin

### 3.1. Liquefaction

**Figure 2:**
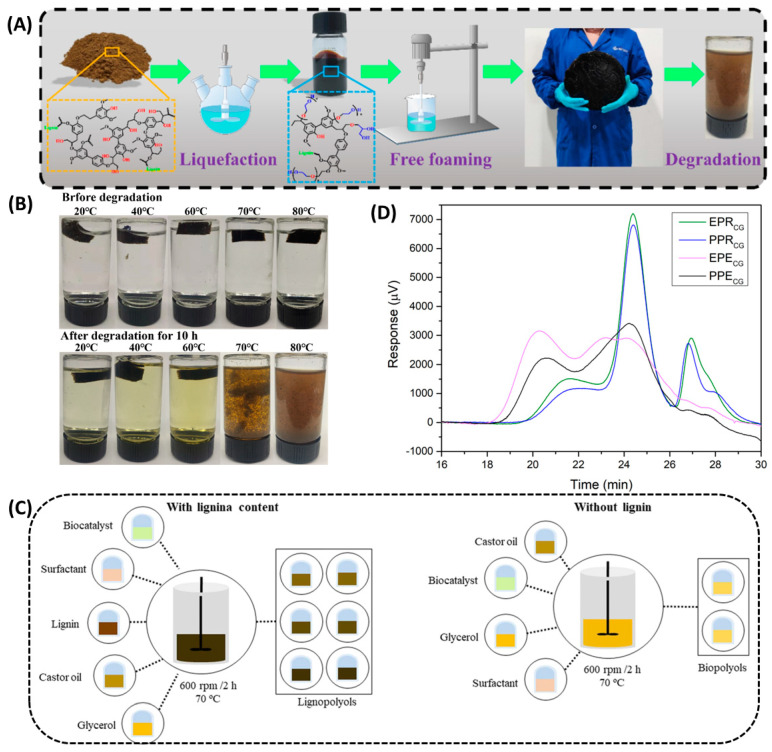
Preparation and properties of lignin-based polyurethane. (**A**) The preparation, scale-up, and degradation of the lignin-based polyurethane foams (the foam is about 30 cm in diameter) [31]. (**B**) The degradation of LPUF30-1 in 2 mol/L NaOH aqueous solution for 10 h at different temperatures, and the corresponding photos of the experiments [31]. (**C**) Illustration of the preparation of lignin and bio-polyols [53]. (**D**) Molecular weight distribution of the liquefied bio-polyols [41].

Liquefaction is one of the most efficient and convenient methods of converting lignin into polyols that can be used in polymeric materials. The biomass is heat-treated under high temperatures with an acid catalyst. This method is usually applied to various wood fiber biomasses such as wood and crop straw [50]. In the liquefaction process, sulfuric acid usually acts as a catalyst, while glycerol and polyethylene glycol (PEG400) act as organic solvents. Using glycerol as a co-solvent can contribute to the liquefaction and convert the reactants into polyether polyols more efficiently. At the end of the reaction, the complex molecules are usually broken into more reactive and smaller molecules through dehydrogenation, decarboxylation, and deoxygenation [51]. Ma et al. [31] successfully prepared lignin polyols using enzymatic lignin digestion with PEG-400 and glycerol as the solvent and sulfuric acid as the catalyst (Figure 2A), and synthesized lignin-based polyurethane foam with excellent photothermal conversion ability. In addition, this study also found that the synthesized PU foams degraded well in alkaline solutions and the rate of degradation increased continuously with increasing temperature (Figure 2B).

Besides utilizing acid catalysts, Feng et al. [52] employed metal-supported mesoporous catalysts for the liquefaction of lignin, achieving a significantly higher yield of phenolic compounds via catalytic liquefaction. Araujo et al. [53] (Figure 2C) used low-sulfur alkaline lignin as the substrate, synthesized bio-polyols through enzyme-catalyzed transesterification, and then obtained high-density rigid polyurethane foam through bulk polymerization. Compared with acid catalysts, enzyme catalysts do not produce many by-products and can be recycled and reused [54].

To improve the sustainability of bio-polyols, the researchers also used microwave-assisted liquefaction to synthesize lignin-based polyols with suitable properties and successfully prepared rigid and elastic polyurethane [41]. At the same time, it has been proved that the crude glycerol recovered from waste oil is suitable for the production of bio-polyols (Figure 2D). To increase the performance of lignin-based polyurethane, Chen et al. [55] modified liquefied lignin polyurethane materials by using the coordinated action of carbon black and polysiloxane, and successfully prepared hydrophobic coating materials by reducing the micropore size of polyurethane materials and improving the hydrophobicity. The polyurethane coating materials showed excellent biodegradability in soil after the modification of lignin.

The liquefaction products of lignin polyols are rich in active hydroxyl groups, with adjustable hydroxyl groups and viscosity, thereby enhancing the reactivity of lignin. The successful liquefaction of lignin also demonstrates its potential for application in high-value-added organic chemicals. The successful liquefaction of lignin also demonstrates its potential for application in high value-added organic chemicals. However, the liquefaction process of lignin usually requires the addition of catalysts, which can lead to different degrees of degradation, condensation, rearrangement, and other side reactions of lignin. Therefore, how to choose the appropriate type and proportion of catalysts to obtain a higher percentage of phenolic compounds is an important research direction in the future.

### 3.2. Alkylation

Alkylation is one of the most common methods of lignin modification, including hydromethylation and hydroxypropyl.

#### 3.2.1. Hydromethylation

To achieve hydromethylation through electrophilic substitution reactions, lignin usually undergoes an addition reaction with formaldehyde under alkaline conditions, introducing hydroxymethyl groups into lignin molecules to enhance the reactivity of lignin. In investigating the hydromethylation of lignin, Hanna et al. [56] found that the complex structure of lignin can cause the condensation of hydroxymethyl groups by simultaneously performing the hydromethylation of phenol and lignin. Therefore, the hydromethylation of lignin requires a strict control of reaction conditions.

**Figure 3:**
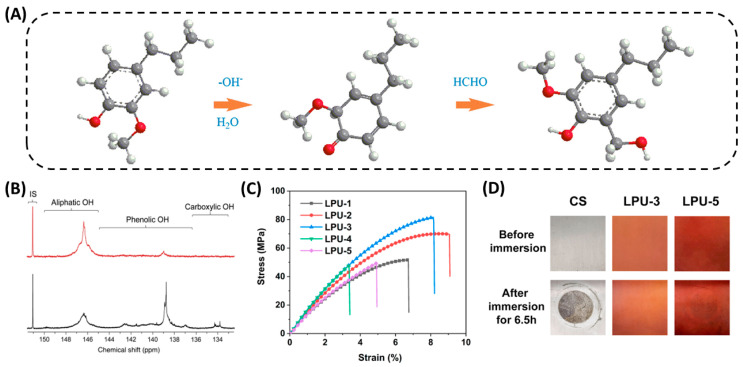
Alkylation modification of lignin. (**A**) Hydromethylation of the phenolic ring of a lignin unit (guaiacol type) [44]. (**B**) 31^P^ NMR spectra of OKL and KL in CDCl_3_ with NHND as an internal standard. Polyalkylation condition of OKL: 60 wt.% PEG400, 20 wt.% lignin loading, 170 °C for 30 min [59]. (**C**) Tensile stress-strain curves of the obtained LPUs [61]. (**D**) Photos of the bare CS and LPU coatings after the short-term corrosion resistance test [61].

Chen et al. [44] performed hydromethylation by mixing lignin acetate in an alkaline formaldehyde solution (lignin/NaOH/HCHO molar ratio of 1/1/2.4) (Figure 3A). The reaction results showed that the optimum reaction temperature for hydromethylation was 85 °C, and the content of hydroxymethyl in the reacted polyol was increased by 189.11% relative to the original lignin acetate. However, there are certain hazards associated with this reaction, as it can lead to a certain amount of formaldehyde remaining in the final product, and the release of free formaldehyde can also be harmful to humans. Therefore, the ratio between formaldehyde and each solution should be strictly controlled to minimize the harm of formaldehyde when using this lignin modification method. In addition, Schieppati et al. [57] used ultrasound-assisted hydromethylation to shorten the reaction time to a certain extent, and the obtained lignin also had good dispersibility. This avoids the high temperature conditions of the reaction.

#### 3.2.2. Hydroxypropyl

Another commonly used method for lignin modification is hydroxypropyl. Under high temperature and pressure conditions, the –OH group on lignin reacts with epichlorohydrin to achieve hydroxypropyl of lignin [58]. The conventional reaction of hydroxypropyl is mainly the conversion of lignin to liquid polyols under high temperature (80–200 °C) and pressure (650–1820 KPa) in the presence of a basic catalyst. Zhang et al. [59] used sulfated lignin (KL) and a mixture of PEG400 and EC (PEG400/EC ethylene carbonate) as co-solvents to initiate the alkoxylation of lignin in a preheated oil bath at 140–170 °C (10–30 min). Phenolic -OH successfully converted to aliphatic -OH, resulting in highly reactive lignin (Figure 3B). However, this method demands high temperatures and sealed reactors, and can lead to the degradation of the lignin. Therefore, more gentle chemical methods are needed to modify lignin and obtain highly reactive polyols. It has been demonstrated that lignin polyols can also be successfully prepared by free radical and photoinduced click reactions, which provide inspiration for the chemical modification of lignin [60]. Cao et al. [61] first selectively converted the phenolic hydroxyl groups on enzymatically digested lignin into aliphatic primary hydroxyl groups via a population reaction, and then used hot radicals to initiate thiol-Ene click reactions to prepare lignin polyols, which could also be used to prepare PU coatings with excellent mechanical properties and high corrosion resistance (Figure 3C,D). Puumala et al. [62] synthesized hydroxypropyl sulfonated lignin in an environmentally friendly manner by reacting kraft paper lignin (KL) with 3-chloro-2-hydroxypropanesulfonic acid (CHPS) under alkaline conditions. The lignin synthesized using this method has good dispersibility and improves the stability of the suspension. This method avoids the use of harsh experimental conditions and also yields a lignin polyol with high reactivity.

In terms of improving the reactivity of lignin with isocyanates, alkylation allows the modification of complex biomass into highly reactive and less hindered compounds by chain extension [63,64,65]. Therefore, it has a high potential for application in the synthesis of polyurethanes. The conventional reaction of alkylation is carried out at high temperature and pressure, which can effectively address the functionalization of hydroxyl groups and convert 100% of the substrate into the final product. In addition, the alkylation of lignin can also be carried out by varying the reaction conditions, such as using microwave-assisted heating as well as catalyst-free conditions, to achieve the maximum conversion of solid feedstock to liquid polyols. In conclusion, for the modification of lignin, each modification has different advantages and disadvantages, but the alkylation of lignin can completely transform the substrate into a highly active reactant, which has a high potential for application.

### 3.3. Acetylation

**Figure 4:**
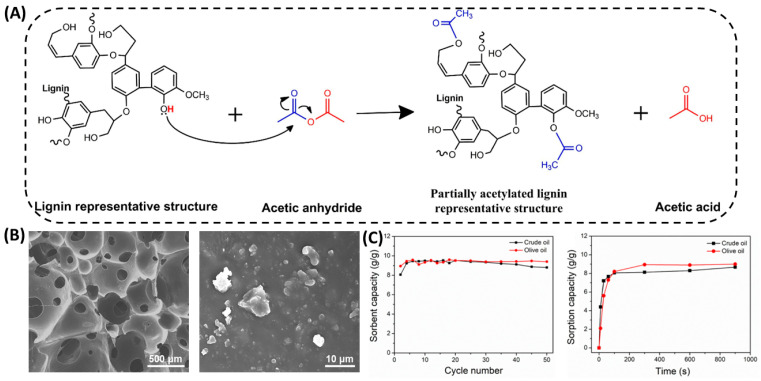
Acetylation modification of lignin. (**A**) Kraft lignin partial acetylation reaction with acetic anhydride scheme [49]. (**B**) SEM images of KLPU foam with KL content of 10 wt.% at different magnifications [66]. (**C**) Recycle number and time, showing reusability and sorption kinetics of the sorbent, respectively [66].

Lignin acetylation is known to be used to improve the properties of lignin, such as solubility, antioxidant, UV-blocking, and the compatibility with other polymers. The degree of lignin acetylation contributes greatly to the value of lignin. The acetylation of lignin usually yields polyols with fewer reactive sites but higher thermal stability. Gouveia et al. [49] conducted a partial acetylation chemical modification of sulfate lignin (Figure 4A), and successfully synthesized lignin-based thermoplastic PU using the obtained lignin macromolecules as polyols. This method allows a controlled reduction in the hydroxyl content of the lignin by half. In addition, Hwang et al. [66] used acetic anhydride to modify lignin sulfate at room temperature. The hydroxyl group of the lignin was replaced by an acetyl group, enhancing the hydrophobic properties of the lignin, and giving the polyurethane foam excellent cycling properties (Figure 4B,C). Shayesteh et al. [67] investigated the effects of reaction temperature, reaction time, and solvent ratio on the rate of lignin acetylation. The results indicate that increasing the temperature from 80 °C to 100 °C will lead to the occurrence of lignin acetylation. However, the use of acetic anhydride reagents has serious drawbacks, such as high cost and sensitivity to water. These drawbacks make it difficult to adopt acetic anhydride in industry. Therefore, it is necessary to search for milder reagents for the acetylation modification of lignin. de Oliveira et al. [68] used acetic acid as an acetylation reagent and employed microwave-assisted partial and selective acetylation of lignin. The results showed that the acetylation reaction only selectively occurred on the aliphatic hydroxyl group while retaining the phenolic hydroxyl group. These studies demonstrated that in order to improve the harsh conditions of lignin modification, the acetylation of lignin moves towards more moderate reaction conditions.

These results indicate that acetylation can selectively position and modify lignin by controlling reaction conditions, which is manageable compared to other modification methods. Compared to traditional esterification reactions using anhydride and acyl chloride, using more environmentally friendly esterification agents can accelerate the acetylation of lignin.

### 3.4. Demethylation

**Figure 5:**
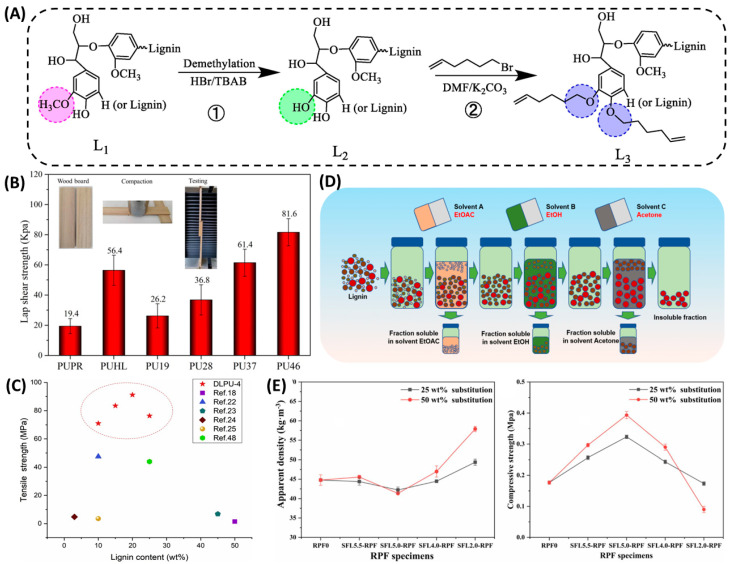
(**A**) ①: Bamboo lignin is demethylated. ②: The L2 lignin is modified by 6-bromo-1-hexene [69]. (**B**) Lap shear strength of PSAs [69]. (**C**) The comparison of tensile strength of PU adhesives among this paper and some studies [39]. (**D**) Steps for sequential solvent fractionation of lignin [29]. (**E**) Mechanical properties of the RPFs, the apparent density, and the compressive strength of the foams [74].

There are reaction sites of aliphatic and aromatic hydroxyl groups in lignin. Demethylation before copolymerization with isocyanate converts the methoxy group to a phenol hydroxyl group and increases its -OH content, which can improve the reactivity of lignin. Xu et al. [69] used renewable lignin to obtain the modified monomer. The phenolic hydroxyl group of lignin increased by 21.4% after demethylation. Furthermore, the shear strength of the polyurethane synthesized from was greatly improved (Figure 5A,B). Studies have shown that laccase can be used as a catalyst to effectively increase the degree of demethylation of lignin, but the lignin obtained has relatively poor reactivity. The use of hydrogen chloride instead of enzyme can effectively increase the number of phenolic hydroxyl groups of lignin and increase the reactivity of lignin. Chen et al. [39] used hydrogen bromide for demethylation under different temperatures and found that the phenolic hydroxyl content of lignin increased by 36.52% at a temperature of 120 °C, producing polyurethane adhesives with higher tensile strength relative to other adhesives (Figure 5C). To explore a milder method, Chen et al. [70] prepared demethylated lignin at room temperature in NaOH/urea solution. The content of phenolic hydroxyl functional groups in the obtained lignin increased by 176.67%, enhancing the reactivity of lignin. Han et al. [71] did not use toxic reagents to prepare lignin-based polyphenols. The content of methoxy groups in the obtained lignin significantly decreased, and there was no degradation or structural change.

However, the process of lignin demethylation reaction is relatively difficult, and the strict control of reaction conditions is necessary to achieve better results. The above research results indicate that increasing the content of phenolic hydroxyl groups in lignin through demethylation can improve the reactivity of lignin and obtain high-value lignin. Compared with original lignin, the antioxidant and thermal stability properties of lignin have also been significantly improved.

### 3.5. Other Methods

In addition to the above methods, lignin can also be chemically modified to prepare high-performance polyurethane materials by fractionation, catalytic depolymerization, and direct utilization. There are various ways for the fractional distillation of lignin to overcome its inhomogeneity and improve its processability, such as the organic solvent method, ultrafiltration, different acid gradient precipitation, and other strategies. Researchers such as Wibowo [72] and Liu [73] sequentially fractionated lignin using ethanol, butanone, methanol, and acetone to obtain four different molecular weights and structures of lignin, and the results showed that the aliphatic hydroxyl content increased gradually with increasing molecular weight. Guo et al. [29] used ethyl acetate, ethanol, and acetone to successively fractionate the lignin to obtain homogenized lignin (Figure 5D), which showed a significant improvement in the compatibility of the lignin with polyethylene glycol and a substantial increase in the mechanical properties of the resulting polyurethane foam. The organic solvent fractionation of lignin can achieve the efficient separation of lignin, cellulose, and hemicellulose, can improve lignin processability, and can effectively increase the overall efficiency of lignocellulose utilization. Furthermore, Cao et al. [74] fractionated lignin samples from corn stover alkali slurry using a gradient acid precipitation method, which resulted in a significant increase in the content of phenolic hydroxyl groups of lignin, showing the potential to replace petroleum resources. The method also revealed a relationship between the hydroxyl content of the lignin polyol and the rigid polyurethane foam, and the resulting polyurethane foam also exhibited high compressive strength (Figure 5E). However, the fractionation of lignin also has drawbacks. When the concentration of the system changes, lignin with different molecular weights will undergo co-precipitation, forming colloids during the precipitation process, which affects the purity and separation efficiency of lignin. The modification of lignin to increase its aliphatic hydroxyl content for high reactivity is an effective strategy in order to improve the high value conversion of lignin fractionation and the efficient substitution of lignin for petroleum resources. Therefore, the choice of grading method should not only take into account the nature and state of the lignin feedstock, but should also include the specific requirements for lignin purity, molecular weight, and dispersibility in the subsequent conversion process.

Although lignin can be fractionated to improve its reactivity, the lower efficiency and application value of lignin fractionation has led to the catalytic depolymerization of lignin gaining interest [75]. The catalytic depolymerization of lignin yields a variety of monophenols that, when stabilized in activity, yield more recalcitrant lignin polymers. In order to improve the efficiency of catalytic depolymerization, different catalysts need to be selected to obtain high yields of monophenols. Liu et al. [76] used poplar chips as raw material to obtain lignin oligomers through the catalytic depolymerization of lignin with 2-PrOH. The results showed that the catalytic depolymerization of crude lignin made it easier to isolate more valuable lignin monomers and use the oligomers as polyol substitutes in the synthesis of polyurethanes. Zeolites were used by Zhang et al. [75] to catalytically depolymerize alkaline lignin to obtain high yields of monophenols for the production of bio-polyurethane foams. It can thus be seen that catalytic depolymerization can also be used to obtain highly reactive lignin, showing a great potential to replace petroleum resources.

In addition to the chemical modification of lignin, lignin bioresources can be extracted from lignocellulosic bioresources waste for partial polyol replacement applications. Gang et al. [77] extracted vanillin from bioresource waste, which can be used as a partial polyol replacement for polyurethane synthesis. The modification of vanillin with monoethanolamide provided structural robustness to the polyurethane. Shi et al. [78] obtained aromatic diesters via selective cleavage from herbaceous lignin using ZnMoO_4_/MCM-41 catalysts. The results showed that the polyurethanes obtained have controlled mechanical, optical, and thermal properties. It can thus be seen that, in addition to the fractionation and depolymerization of lignin, it is also possible to use bioresource waste to obtain low molecular compounds to replace petroleum resources, and the polyurethane materials obtained show good potential for development.

At present, the use of lignin suffers from difficulties in isolation, and it is not yet possible to fully characterize the structure of lignin accurately [79]. Consequently, there are difficulties in the direct utilization of lignin when homogeneous lignin cannot be completely prepared. In addition, chemically modified lignin has increased economic and natural costs due to the use of a large number of chemical reagents and the complexity of the treatment process. At the same time, the lignin modification process can effectively improve the lignin hydroxyl content, but is generally designed to use expensive catalysts, high temperature and high pressure, and strong acid and alkali conditions, and is not suitable for large-scale industrial production. Therefore, there is an urgent need to develop environmentally friendly and efficient separation technologies and to improve the production efficiency of lignin-derived platform chemicals, so as to provide lignin-based polymer materials with a wide variety of high-quality starting materials.

## 4. Application of Lignin-Based Polyurethane in Flexible Electronics

The addition of lignin improves the properties of lignin-based polyurethanes such as thermal stability, UV resistance, and degradability. These can effectively improve the functionality and durability of flexible electronic devices. At present, lignin-based polyurethanes are often used in the form of films, elastomers, foams, and gels to enhance the mechanical properties and multifunctionality of electronic materials. It can be compounded with a variety of electronic materials to obtain high-performance electronic devices.

### 4.1. Polyurethane Films

Polyurethane films are widely used in healthcare, industry, and everyday life due to their excellent abrasion resistance, breathability, and biocompatibility. In addition, replacing some polyols with lignin to synthesize polyurethane can not only improve its mechanical properties but can also endow it with additional properties. These results can be applied to more aspects of polyurethane.

**Figure 6:**
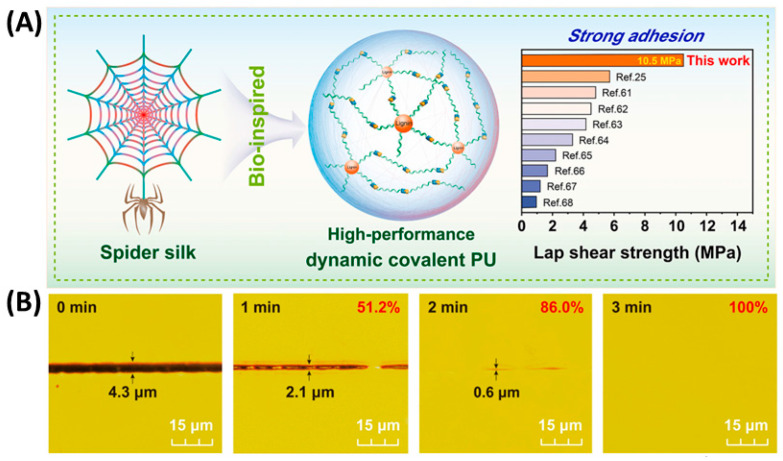
(**A**) Schematic diagram of the spider silk spatial hierarchy [18]. (**B**) Optical images of MLPU-2 film at different repairing times at 80 °C [18].

**Figure 7:**
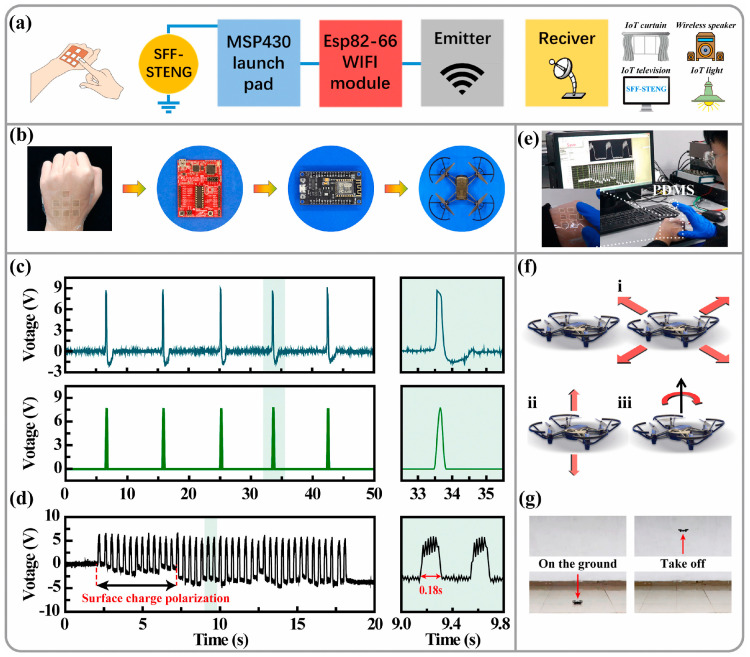
Application of tactile sensing based on the SFF-STENG in the Internet of Things by WLAN [83]. (**a**) Working diagram of a tactile sensing control system. A press signal trigged by tactile sensing can control the appliances by some electric modules. (**b**) Optical photographs of components of the tactile sensing control system for the drone movement control. (**c**) The SFF-STENG generates original triboelectric voltage input and output signal after rectification. (**d**) Voltage waveform diagram of a tactile sensor based on the SFF-STENG by fingertips touch. (**e**) Tactile sensing test based on SFF-STENG by fingertips press. (**f**) Tactile signal for the drone movement in (i) forward/backward/left/right direction, (ii) up/down direction, and (iii) right rotation. (**g**) Diagram of the drone movements.

Xue et al. [80] successfully prepared lignin-based polyurethane films by using modified alkaline lignin instead of partially polycarbonate glycol (PCDL) for casting. The research results indicate that the polyurethane film added with lignin has a significant UV-blocking effect, and the material performance of lignin-based polyurethane is the best when the R-value is 1.8. Zheng et al. [18] (Figure 6A,B) modified lignin using a one-step method as a cross-linking agent to prepare high-performance modified lignin-based dynamic covalent polyurethanes (MLPUs). This polyurethane film has high repair efficiency and excellent shape memory ability. As the same time, Yang [45] and others used modified lignin in the synthesis of polyurethane films. Studies have shown that lignin-based NIPU has a higher tensile strength than other reported tensile strengths, while imparting antioxidant and UV-resistant properties and also possessing antimicrobial properties. To enhance the application value of polyurethane, many researchers enhance its performance by compounding it with other materials. Wu et al. [81] prepared an environmentally friendly closed lignin-based polyurethane lotion through pre-polymerization, which was used to prepare a biodegradable regenerated-fiber composite membrane. The composite film exhibits excellent UV barrier properties and good thermal stability. Wang [82] et al. used polyurethane foam, a polymer waste material, as a substrate to prepare flexible electrodes by encapsulating carbon nanotubes and cellulose nanoparticles. In addition, the polyurethane substrate material can also be hot-pressed to produce a flexible pure polyurethane film, which can be used to encapsulate electrodes to form an energy wristband to power an electronic watch. This research not only enables the use of polyurethane for wearable electronic devices but also the rational use of polymer waste. In addition to being a base material, polyurethane can also be used as a functional material. Researchers such as Gong [83] functionalized silk protein with the addition of glycerol and polyurethane to improve the flexibility of silk protein membranes. By constructing hollow silver nanofibers on the silk protein membrane, a breathable, stretchable, and degradable thin layer was formed for use as a friction electrode (Figure 7). Lignin-based polyurethanes have also shown great potential in human health monitoring. Researchers such as Ma prepared lignin-based covalently adaptive polyurethane fabrics (LPUF) containing graphene composite [84]. The study demonstrated that LPUF-20 was able to accurately detect not only large deformation signals, but also capture small deformation signals, such as respiratory vibration and respiratory airflow. In addition, the fabric prepared in this study not only has a unique advantage in breathability, but also exhibits solvent resistance due to the cross-linked network, which can be used to fabricate solvent-resistant wearable sensing devices.

These studies indicate that replacing polyols with lignin can effectively improve the mechanical properties of polyurethane. Meanwhile, the composite of polyurethane materials can also be applied to a wider range of fields. The addition of lignin gives the polyurethane film excellent UV resistance [85], which not only protects the device from the sun’s UV rays, but is especially important for wearable devices used outdoors. This property of lignin significantly improves the durability and extends the life of the device. Furthermore, bio-based polyurethane film can enhance the mechanical strength and stability of flexible electronic substrates, and has biocompatibility and breathability, which can better fit the human skin and be better applied in the field of flexible electronic devices.

### 4.2. Polyurethane Foams

Polyurethane foam is a porous polyurethane material consisting of a large number of microscopic pores, whose main characteristics are porosity, low density, and relatively good permeability and insulation [46,86]. They are one of the most-used polyurethane materials. Lignin-based polyurethane foams can improve their mechanical properties and are also applied to multiple fields; therefore, lignin-based polyurethane foams are also of great interest to researchers.

**Figure 8:**
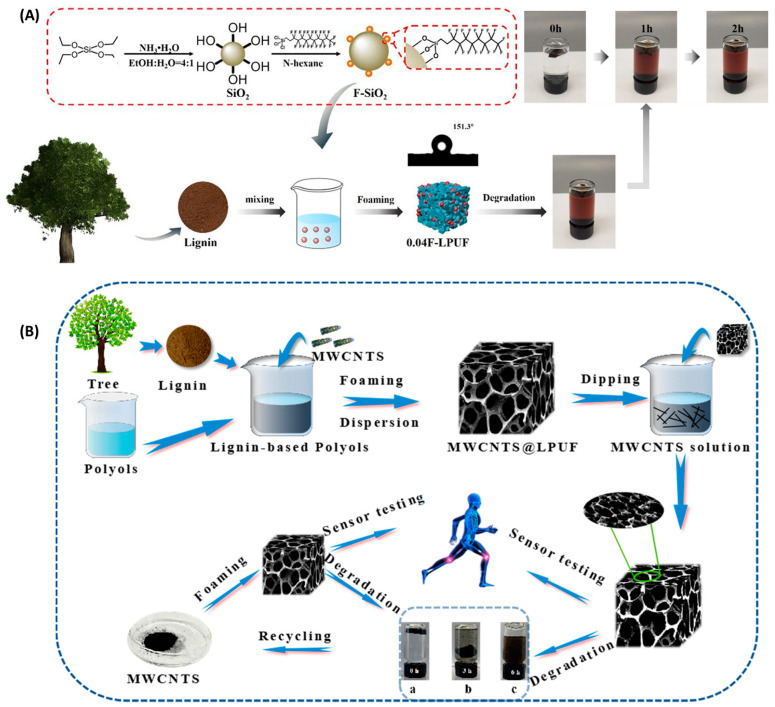
(**A**) Schematic representation of the preparation and degradation of superhydrophobic 0.04F-LPUF foam adsorbent and photograph of 0.04F-LPUF degradation over time in aqueous sodium hydroxide solution in methanol [88]. (**B**) Schematic illustration of the fabrication process of the MWCNTs @ LPUF sensor [93]. (**a**–**c**) Degradation of MWCNTs @ LPUF sensors at 0–3 h.

At present, the most commonly used field of lignin-based polyurethane is oil–water separation. Lv et al. [87] used lignin and castor oil as alternative resources of petroleum-based raw materials to synthesize biodegradable polyurethane foam for oil spill treatment. Chen et al. [40] used a one-step foaming process to synthesize lignin polyurethane foam, and at the same time used phytic acid to modify foam to achieve the characteristics of having a hydrophilic surface and being hydrophobic underwater. In addition, researchers such as Wu [88] also added SiO_2_ modified with 1H, 1H, 2H, and 2H-perfluoro dodecyl trichlorosilane (F-SiO_2_) to a lignin-based foam matrix in one step to obtain polyurethane foams with hydrophobic properties (Figure 8A). The above results show that the mechanical properties of polyurethane foam have been greatly improved with the addition of lignin, and the polyurethane foam also has good biodegradability in an alkaline environment.

In addition to its application in sewage treatment, biological foam with more properties can also be obtained through compounding. Li et al. [89] synthesized lignin-based polyurethane foams by enzymatically promoting the alkylation and cyclic carbonation modification of lignin while adding silver nanoparticle solutions to confer antimicrobial properties during the foaming process. By observing in vitro wounds in mice, it was shown that this antimicrobial composite foam could rapidly promote wound healing and repair damaged tissues. This also suggests that this bio-based biodegradable antimicrobial foam has great potential for clinical applications in wound management. Cui et al. [90] compounded lysine (PL), Ag NPs, and lignin-based polyurethane to obtain composite biological foam. The research results show that biological foam can be used as a fast (within 1 h) and effective antibacterial wound dressing for Gram-positive Staphylococcus aureus and Gram-negative Escherichia coli. Li et al. [91] prepared lignin-based polyurethane foam by replacing petroleum raw materials with bio-based polyether polyols. At the same time, the composite of the foam with Ag also showed better mechanical and antibacterial properties. This new super hydrophobic and antibacterial composite biological foam from natural lignin-based polyurethane has great advantages in medical dressings and can be used for antibacterial disinfection and the repair of damaged tissues in future clinical practice. The antimicrobial properties of lignin protect wearables from bacteria, which is especially important for wearables that are in prolonged contact with the skin. The addition of lignin also improves its antimicrobial properties, which reduces the risk of bacterial infections and safeguards the user’s health.

Furthermore, Carrico et al. [92] synthesized semi-rigid polyurethane foams from a physical mixture of sulfated lignin, castor oil, and residual glycerol. The results showed that the thermal stability of foam with higher lignin content decreased. Researchers such as Zhang [59] used sulfated lignin (KL) to create biological polyols to produce rigid polyurethane (RPU) foam, but excessive lignin also led to the decline of polyurethane foam performance. Cao et al. [74] synthesized rigid polyurethane foam (RPF) using maize straw as the main lignin-extracting substance. Studies have shown that the content of β–O–4 bonds in the lignin structure is the main factor influencing the thermal conductivity of RPF and that the higher hydroxyl content of lignin increases the compressive strength of polyurethane foams, leading to a reduction in their apparent density. It can be seen that bio-based polyurethane foams can improve the mechanical properties and stability of the foam, but it is also important to select the most suitable lignin content so that the application areas of lignin-based polyurethane foams can be expanded. In addition, lignin-based polyurethane foams can also be used in health monitoring applications. Wang et al. liquefied lignin into lignin polyol and used a foaming method to prepare lignin-based polyurethane foam containing multi-walled carbon nanotubes (MWCNTs) in one step [93] (Figure 8B). The study shows that the sensor can not only detect a wide range of limb and joint movements, but can also detect small physiological signals such as coughing, swallowing, and breathing; meanwhile, the sensor can record the current changes in respiration and pulse very well, and the results show that the sensor has the potential to be used in medical and healthcare applications.

The above studies indicate that using lignin to replace some polyols can improve the mechanical properties of polyurethane and can be applied in the field of flexible electricity. Therefore, the development of high-performance, recyclable, and stable polyurethane foam can improve the durability of flexible electronic equipment. We also believe that the bio-based polyurethane foam has extensive application potential in flexible electronic equipment.

### 4.3. Polyurethane Elastomers

High-performance polyurethane elastomers offer excellent healing and recovery capabilities, resistance to damage, and high strength and toughness. They are also expected to find their way into wearable electronic devices. However, the stable covalent cross-linking network in these elastomers is irreversible and is often subject to aging, wear, and spontaneous fracture in service, while the introduction of dynamic bonding (dynamic covalent and non-covalent bonding interactions) can be effective in preparing self-healing and reusable polyurethane elastomers [94]. The main objective of using lignin-modified polyurethane elastomers is to reduce costs and improve their properties such as aging and strength.

**Figure 9:**
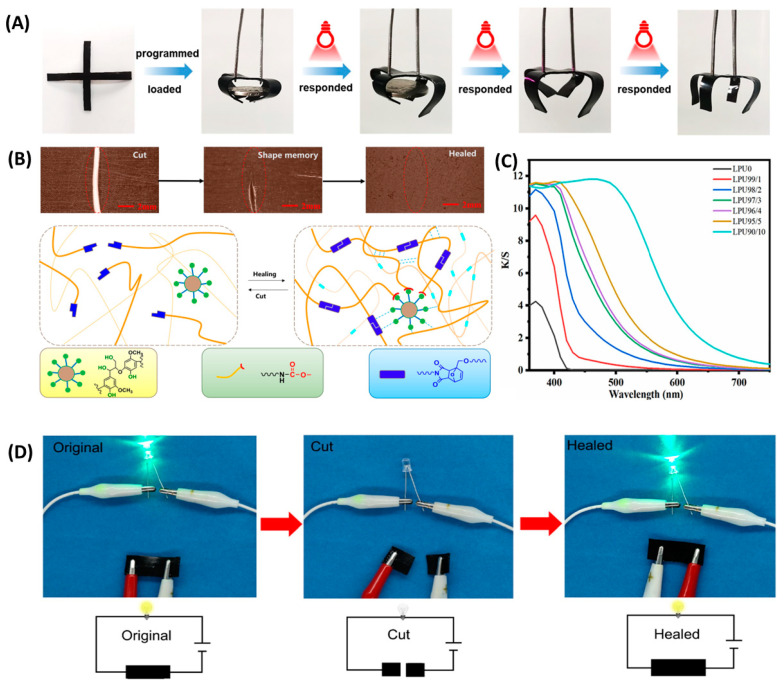
(**A**) Shape memory effect based on the photothermal conversion capability of LPUs [95]. (**B**) Self-healing process of lignin self-healing polyurethane [96]. (**C**) Ultraviolet transmittance curve [98]. (**D**) Photographs showing the restoration of a closed conduction circuit containing an LED after PU-NM1-CNT3 cutting and healing (the illustration is a schematic diagram of the circuit) [100].

The synthesis of lignin-based polyurethane elastomers usually involves introducing covalent hydrogen bonds to enhance their properties. Huang et al. [95] prepared high-strength, heat-resistant, and recyclable lignin-based polyurethane by introducing weak hydrogen bonds, strong hydrogen bonds, and dynamic silane ether covalent bonds. The research results indicate that the abundant conjugated structure of lignin endows LPUs with excellent photothermal conversion ability (Figure 9A). Du et al. [96] substituted hydroxylated lignin with cross-linking agents to synthesize polyurethane materials with self-healing properties. The results indicate that the network structure formed between lignin and DA bonds through the dynamic cross-linking of hydrogen bonds gives polyurethane good thermal stability and self-healing ability (Figure 9B). Yang [97] and other researchers prepared polyurethane elastomers by introducing hydrogen bonds through 2-ureido-4[1H]-pyrimidine (UTCNF)-modified cellulose oxide nanofibers (TCNF) and disulfide bonds (SS) into the polyurethane (PU) backbone to form a double dynamic cross-linked network. It was shown that the elastomer could be completely self-healing at 50 °C while achieving reinforcing and self-toughening properties after multiple hot-pressings. The above research results all indicate that dynamic covalent bonding can effectively enhance the performance of polyurethane. In addition, Li et al. [98] synthesized the hydroxyl structure of polyurethane macromolecules using lignin macromolecules as raw materials, resulting in lignin-based polyurethane with excellent transparency and UV resistance (Figure 9C).

To obtain high-performance composite materials, many researchers use environmentally friendly biodegradable resources to synthesize polyurethane or add other biological resources (cellulose, lignin, chitosan) to obtain high-strength composite materials, which is very important. Huang et al. [99] reported a preparative solvent-free method for the preparation of high-performance lignin-containing polyurethane elastomers, where the free hydroxyl groups of lignin react with isocyanates to form a strong chemical cross-linked network and the unreacted hydroxyl groups form a dynamic hydrogen-bonded network with the polyurethane matrix, forming a double cross-linked network structure with an excellent reprocess ability and aging resistance. The above also reflects the fact that the addition of lignin improves the antioxidant properties of polyurethane, which helps to resist oxidative stress and prevents the deterioration of the equipment material. This is critical to maintaining equipment performance and extending service life.

Researchers such as Dong et al. [100] have developed a self-healing and recyclable conductive composite material with good self-healing and mechanical properties such as a flexible strain sensor using polyurethane (PU) elastomer containing dynamic borate as the polymer matrix and carbon nanotubes (CNT) as the conductive filler (Figure 9D). Gang et al. [77] also synthesized bio-based polyurethane using lignin biological resources. Research has shown that the mechanical properties of the prepared polyurethane elastomer increase with the increase in lignin content.

These studies all indicate that polyurethane can be modified using biomaterials, which is more in line with the goal of sustainable development. The development of highly self-healing polyurethane elastomers significantly extends the service life of the corresponding engineered products and contributes to global economic and sustainable development. We also believe that such self-healing polyurethane elastomer materials also have the potential for applications in wearable electronic devices such as electronic skins and health testing.

### 4.4. Others

**Figure 10:**
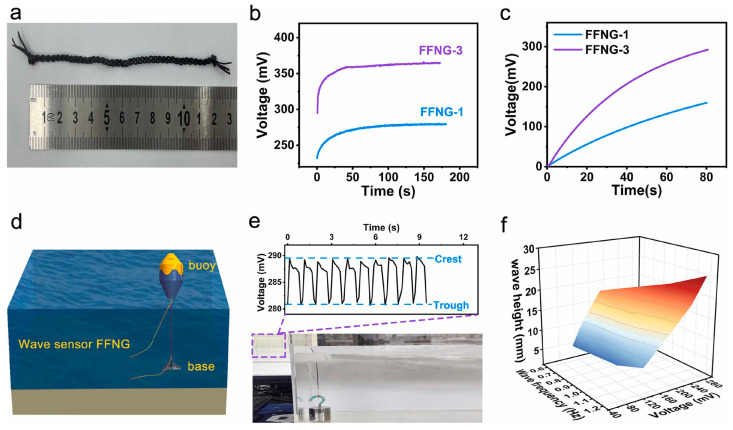
Instantaneous output voltage signal of several wave periods obtained from the FFNG-based wave sensor and instantaneous wave height versus wave frequency and output peak voltage [102]. (**a**) Photograph of a 12-cm-long FFNG woven from three T40/L60 fibers (**b**) The output voltage generated by FFNG-1 and FFNG-3 with a contact length of 6 cm in 0.6molL-1 NaCl solution. (**c**) Plots of charged voltages across a 10 μF capacitor in FFNG-1 and FFNG-3. (**d**) Schematic diagram of the ocean wave monitoring device. (**e**) Instantaneous output voltage signal of several wave periods obtained from the FFNG-based wave sensor. (**f**) Instantaneous wave height versus wave frequency and output peak voltage.

In addition to the above-mentioned applications of lignin in polyurethane, some researchers have also used lignin in research areas such as the preparation of polyurethane hydrogels, water-based polyurethanes through modification, and nanogenerators [101]. Gao and other researchers [102] have prepared a strong, flexible and stretchable fibrous fluidic nanogenerator (FFNG) using carbonized lignin and thermoplastic polyurethane via wet spinning. The study showed that the device can achieve high direct current (DC) voltages of up to 420 mV. It can also act as a self-powered wave sensor, providing basic information about wave conditions (Figure 10).

**Figure 11:**
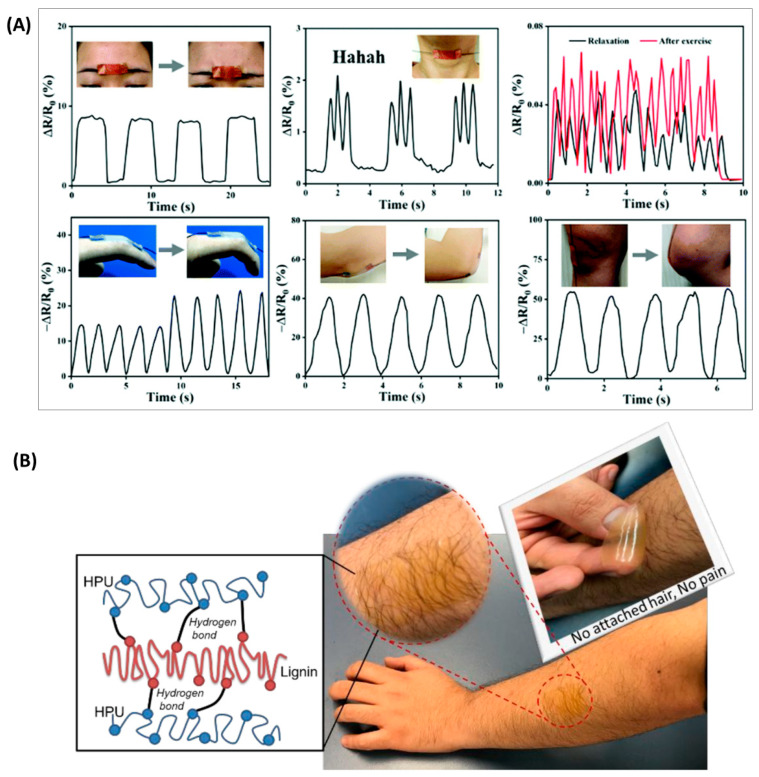
Lignin-based polyurethanes for wearable flexible electronics applications. (**A**) Detection of human physiological signals: frown; say Hahaha; pulse at relaxation and excitation; finger; elbow; knee [103]. (**B**) Peeling off the hydrogel film without any attached hair and without any pain [104].

In addition to being a generator, it can also be used as a hydrogel for flexible electronics applications. Wang et al. [103] constructed a multifunctional ionic gel based on Ag–lignin nanoparticles (Ag–lignin NPs), polyurethane (PU), and ionic liquid with excellent self-healing properties through the introduction of disulfide bonds. The multifunctional hydrogel also has excellent antibacterial and UV absorption capabilities due to the introduction of Ag–lignin nanoparticles. As this ionic hydrogel also has excellent sensing stability, it has promising applications in the field of human movement and personalized physiological health monitoring (Figure 11A). By introducing lignin as the toughening part of the polyurethane, Oveissi et al. [104] obtained polyurethane hydrogels with a threefold increase in tensile strength and an increase in elongation at break, from 150 to 240%. In addition, the lignin-added polyurethane hydrogels were also easily processed using fiber spinning, casting, and 3D printing and were biocompatible with primary human dermal fibroblasts (Figure 11B). In addition to the above applications, lignin-based polyurethanes can also be used as electron shielding materials. Hu et al. [105] have introduced lignin with modified carbon nanotubes (CNT) and aminated ferric oxide nanoparticles (Fe_3_O_4_) as reinforcements in polyurethane materials to improve their electron shielding effect.

In summary, lignin-based polyurethanes are used in a variety of applications. Compared to other biomaterials, lignin has a lower raw material cost, making lignin-based polyurethanes more cost-effective. At the same time, lignin-based polyurethanes are typically lightweight and exhibit good elasticity and strength as a flexible substrate, which can improve the comfort and long-term use of wearable devices [91]. In addition, lignin-based polyurethanes can be chemically modified to introduce a variety of functional groups that can impart specific properties to the material, expanding its application in wearable devices. However, lignin-based polyurethanes also face great challenges in wearable electronic devices. The chemical modification of lignin makes it difficult to achieve the standardization and consistency of properties due to the uncertainty of the reaction conditions. Secondly, lignin-based polyurethanes typically have poor electrical conductivity and need to be modified to meet the requirements. This modification process may affect other properties of the material and increase costs. The economics of lignin-based materials compared to traditional petroleum-based polyurethanes remains a challenge. Despite the sustainability and environmental benefits of lignin-based polyurethanes, their specific application scenarios in wearable electronics are not yet fully defined. Therefore, expanding the use of lignin-based polyurethanes in flexible electronic devices still faces significant challenges.

## 5. Conclusions and Perspective

Due to the low reactivity of lignin,, most of them need to be chemically modified to achieve the green synthesis of lignin. This is one of the difficulties faced in their application. To achieve their wider application, bio-based polyurethanes are being developed for wearable electronic devices to provide a more comfortable experience for the human body. Lignin shows great potential as a new green material, due to its special properties such as antioxidant properties, UV resistance, antibacterial, and stabilization properties, making this biomaterial promising for wearable electronic uses. However, there are still some challenges in the wider application of lignin due to its complex chemical structure depending on the source of the lignin. To overcome these limitations, several methods have been proposed to modify lignin and increase the number of phenolic hydroxyl groups in lignin polyols through methods like fractionation, catalytic depolymerization, acylation, liquefaction, and alkylation, thereby increasing the reactivity between lignin itself and isocyanates and improving the efficiency of polyurethane synthesis.

Most of the available research focuses on the extraction process and chemical modification of lignin and on polymers that have been developed with a biological base. This review discusses the modification of lignin-synthesized polyurethanes as a broad class of polymers and the development of greened polyurethanes synthesized from biological resources for use in flexible electronic devices. Due to the unique properties of lignin itself, the application of flexible electronic devices has great potential in the wearable sector. Furthermore, most of the applications of lignin-based polyurethanes in wearable electronic devices are used for detecting human activities and skills, and are used less in applications such as health monitoring. Therefore, given the chemically modified lignin, the synthesis of greened polyurethanes and their growth potential for applications in wearable electronic devices is expected to be an important area of research soon.

In summary, future research aspects should further improve the efficiency of lignin polyols in polyurethane synthesis reactions, while confirming that lignin-based polyurethanes can be used as flexible substrates for a wider range of applications in health monitoring, in addition to having more applications in flexible electronic devices.

## Figures and Tables

**Table 1:** Properties of lignin-based polyurethanes.

Preparation Method	Material	Description of the Preparation Method	PU Properties
Liquefaction	Enzymatically hydrolyzed lignin (EL), Polyethylene glycol 400 (PEG-400), Glycerol, Hexamethylene diisocyanate (HDI).	PEG-400 and glycerol as co-solvents.	Excellent mechanical stability, degradability [40].
	Eucalyptus globulus, PEG-400, Glycerol, H_2_SO_4_ (Sulfuric acid).	PEG-400, glycerol, and sulfuric acid as co-solvents.	Increased tensile strength and elongation at break [41].
	Bamboo, Methanol, H_2_SO_4_, Methylene Diphenyl diisocyanate (MDI).	Liquefaction using a microwave.	Improved thermal stability, used for building insulation [42].
	Bamboo, Methanol, H_2_SO_4_, MDI.	H_2_SO_4_ as a catalyst, liquefaction using a microwave.	Excellent flame retardancy, insulation properties, and thermal stability [43].
hydromethylation	Acetic acid lignin (AAL), Formaldehyde, PEG-200, Toluene-2,4-diisocyanate (TDI).	Lignin can chemically react with formaldehyde.	Enhanced mechanical properties, thermal stability, and tensile strength [44].
hydroxypropyl	Sawdust, Epichlorohydrin (ECH), Isophorone diamine (IPDA), DMF.	Fractionated oligomers for hydroxypropyl.	Excellent heat stability, antimicrobial properties, UV resistance [45].
	Rapeseed oil-based polyol (RP), Lignin-based polyol (LP), Peracetic acid.	Fractionated oligomers for hydroxypropyl.	Improved thermal stability and degradation properties [46].
	Rapeseed oil-based polyol (RP), Lignin-based polyol (LP), Peracetic acid.	Fractionated oligomers for hydroxypropyl.	Excellent thermal conductivity, thermal insulation materials [47].
	Rapeseed oil-based polyol (RP), Lignin-based polyol (LP), Peracetic acid.	Fractionated oligomers for hydroxypropyl.	Excellent heat stability and compressive strength [48].
Demethylation	AAL, DMF, Hydrobromic acid, PEG, TDI.	Catalytic demethylation of lignin by heating with HBr in DMF solvent.	Rubber-like tensile properties and improved thermo-oxidative stability [39].
Acetylation	Eucalyptus wood (KL), PEG, Acetic anhydride, Tetrahydrofuran (THF), MDI.	React lignin with Acetylation at 80 °C for 48 h.	Enhance modulus of elasticity and excellent bonding properties [49].
Fractionation	Alkaline lignin, Ethyl acetate (AR), Ethanol, Hydrochloric acid, PEG, HDI.	Fractionated lignin using ethyl acetate, ethanol, and acetone.	Improved the mechanical properties [29].
